# Long-Term Visual Outcome in Wet Age-Related Macular Degeneration Patients Depending on the Number of Ranibizumab Injections

**DOI:** 10.1155/2015/820605

**Published:** 2015-09-27

**Authors:** Pilar Calvo, Beatriz Abadia, Antonio Ferreras, Oscar Ruiz-Moreno, Jesús Leciñena, Clemencia Torrón

**Affiliations:** ^1^Ophthalmology Department, Miguel Servet University Hospital, Isabel la Catolica 1-3, 50009 Zaragoza, Spain; ^2^University of Zaragoza, Zaragoza, Spain

## Abstract

*Purpose*. To analyse the visual outcome in wet age-related macular degeneration (AMD) patients depending on the number of ranibizumab injections.* Methods*. 51 naïve wet AMD patients were retrospectively recorded. Visual acuity (VA), central retinal thickness (CRT) measured with spectral domain (SD) optical coherence tomography (OCT), and number of intravitreal injections were compared at 6, 12, 18, 24, 30, and 36 months of follow-up. Kaplan-Meier survival rates (SRs) based on VA outcomes were calculated depending on the number of ranibizumab injections performed.* Results*. VA improved compared with baseline at 6 and 12 months (*P* < 0.005). No differences were found at 18, 24, 30, and 36 months (*P* > 0.05). CRT measured with Cirrus OCT decreased (*P* < 0.001) at all time points analysed. The mean number of injections received was 6.98 ± 3.69. At 36 months, Kaplan-Meier SR was 76.5% (the proportion of patients without a decrease in vision of more than 0.3 logMAR units). VA remained stable (≤0.01 logMAR units) or improved in 62.7%. Within this group, SR was 92.9% in those who received 7 or more injections versus 51.4% receiving <7 treatments (*P* = 0.008; log-rank test).* Conclusion*. Better VA outcomes were found in stable wet AMD patients after 3 years of follow-up if they received ≥7 ranibizumab injections.

## 1. Introduction

Intravitreal antivascular endothelial growth factor (anti-VEGF) agents are the treatment of choice for wet age-related macular degeneration (AMD). Multicentre studies such as the Minimally Classic/Occult Trial of the Anti-VEGF Antibody Ranibizumab in the Treatment of Neovascular AMD (MARINA) and Anti-VEGF Antibody for the Treatment of Predominantly Classic Choroidal Neovascularization (CNV) in AMD (ANCHOR) [1, 2] have shown that monthly intravitreal injections of ranibizumab over a 2-year period not only maintained but also improved best corrected visual acuity (BCVA), with a mean gain of 7.2 and 11.3 letters, respectively.

However, most centres have considerable difficulty maintaining this type of treatment schedule due to an ever-increasing patient load and stress on the patient and family members to attend monthly assessments, as well as the associated economic costs. Alternate regimens of treatment have evolved in an attempt to provide comparable results in terms of BCVA, but with a fewer number of injections.

The PIER and EXCITE trials, using a regimen of three monthly injections as a loading dose and then quarterly injections, failed to match the good results of BCVA [3, 4]. On the other hand, those based on three initial monthly injections followed by an as-needed decision to treat (PrONTO, SUSTAIN) obtained very similar BCVA results averaging fewer injections [5–7].

In MARINA [1], ANCHOR [2], and PrONTO [6] trials, 94.6%, 96.4%, and 97.5% of patients prevented the loss of 15 letters (3 lines) BCVA at 2 years of follow-up. In patients who remained stable (with 0 or more earned letters), BCVA was 71%, 78%, and 78%, respectively.

The purpose of this study was to conduct a survival analysis based on the results of the BCVA and the number of intravitreal injections in wet AMD patients treated exclusively with ranibizumab and 3 years of follow-up in normally run vitreoretinal practices, reflecting a “real-life” clinical setting.

## 2. Methods

### 2.1. Study Design

It was analytical, observational, longitudinal, retrospective study.

### 2.2. Inclusion Criteria

Inclusion criteria include

1. patients >50 years of age;
2. choroidal neovascularization (CNV) due to AMD diagnosed by fluorescein angiography (FA) with at least 3 years of follow-up, with lesion size no more than 5400 *μ*m in greatest linear diameter in the study eye;
3. presence of subretinal fluid (SRF), intraretinal fluid (IRF), or central retinal thickness (CRT) >250 microns on spectral domain (SD) optical coherence tomography (OCT);
4. BCVA of 20/40 to 20/400 (Snellen equivalent determined with the use of a Snellen chart).

### 2.3. Exclusion Criteria

Exclusion criteria include

1. use of any other antiangiogenic medication other than ranibizumab;
2. previous treatment with verteporfin photodynamic therapy, intravitreal steroids, or laser;
3. any history of uveitis, diabetic retinopathy, or any other retinal disease other than AMD.

### 2.4. Population

In this cohort, initial treatment consisted of a loading dose of three intravitreal injections of ranibizumab (0.5 mg) at monthly intervals. One month after the third injection, patients underwent a full ophthalmic examination including BCVA, biomicroscopy, and OCT. Disease state was deemed to be still active if BCVA was worse by 5 letters or if there was any clinical or OCT evidence of disease activity (haemorrhage, SRF, or IRF). If this was the case, patients were given another injection of ranibizumab and were instructed to return in one month for another full clinical and OCT evaluation. This was repeated on a monthly basis until disease state was deemed inactive.

Disease state was deemed to be inactive if BCVA was stable or improved and there was no clinical or OCT evidence of disease activity (hemorrhage, SRF, or IRF). If this was the case, ranibizumab injections were suspended, and patients were instructed to return on a monthly basis for a full ophthalmic examination including BCVA, biomicroscopy, and OCT.

If during the patient's monthly examination there was either a 5-letter decrease in BCVA or clinical or OCT evidence of hemorrhage, IRF, or SRF, the patient was deemed to have developed a recurrence of active wet AMD. Monthly injections of ranibizumab were reestablished until the disease state was deemed inactive. If the disease state became inactive again, ranibizumab treatment was suspended again, and the patient was monitored with clinical and OCT examination on a monthly basis until there was a recurrence.

### 2.5. Study Procedures

The study protocol was approved by the Clinical Research Ethics Committee of Aragón (CEICA), Zaragoza, Spain.

We retrospectively analysed the consecutive charts and FA of three vitreoretinal practices (OR, JL, and CT) at the Miguel Servet University Hospital (Zaragoza, Spain) in patients with naive CNV secondary to AMD who had been treated exclusively with ranibizumab from the first of October 2008 to the first of October 2012.

Baseline data was collected: BCVA, age, gender, slit lamp examination of the anterior segment, intraocular pressure (IOP), dilated fundus examination, FA, and OCT.

The following data was also collected on a quarterly basis: BCVA, CRT measured by OCT, and number of ranibizumab injections.

Snellen visual acuity (VA) was measured by a certified ophthalmic technician. The Snellen value was recalculated to determine the corresponding logarithm of the minimum angle of resolution (logMAR) value for statistical analysis using a formula that reflects the relationship between the two methods [8].

CRT was determined using SD Cirrus OCT (Carl Zeiss Meditec, USA). Scanning with the Cirrus OCT was performed with the 512 × 128 scan pattern where a 6 × 6 mm area on the retina was scanned with 128 horizontal lines, each consisting of 512 A-scans per line (with the total of 65,536 sampled points) within a scan time of 2.4 seconds. All scans were performed by an experienced ophthalmic technician. Only good-quality examinations with a signal strength of ≥6/10 were retained.

### 2.6. Statistical Analysis

All statistical analyses were performed using IBM's statistical software (SPSS version 19.0; IBM Corporation, Somers, NY).

BCVA and CRT changes were compared every 6 months until the end of the study follow-up: 6, 12, 18, 24, 30, and 36 months (matched *t*-test).

The main statistical study was a survival analysis, which represents time-to-event data. Subjects were followed over time and observed at the time point at which they experienced the event of interest.

In this study, 2 independent survival analyses were performed: (i) the first event was considered to be a worsening of BCVA (defined as a difference of >0.3 logMAR units or more from baseline) and (ii) the second event was stable or improving BCVA (defined as ≤0.01 logMAR units compared with the baseline). Survival analysis depending on the mean number of ranibizumab injections (cut-off point of 7) was also performed.

Censoring occurred either when the patient attained each event or at the end of the study period (3 years). Differences in the Kaplan-Meier survival plots were calculated by the log-rank test. For all analyses, *P* < 0.05 was considered statistically significant.

## 3. Results

Of the 122 charts reviewed, 51 eyes (51 patients) met the study inclusion criteria. Baseline demographic and ocular data for both study groups are summarized in Table 1. Mean age was 79.5 ± 7.8 years. Women comprised 58.8% of the cohort, 60.8% of the patients had high blood pressure, and the right eye was treated in 49% of the cases. FA revealed a CNV distribution that was 48.2% predominately classic, 7.4% minimally classic, and 44.4% occult. Locations of these lesions were 31.8% subfoveal, 40.9% Juxtafoveal, and 27.3% extrafoveal. The mean baseline logMAR BCVA was 0.68 (20/95) ± 0.23 and mean CRT by OCT was 342.12 ± 121.57 *μ*m. Mean IOP was 16.16 ± 2.78 mmHg.

Changes in BCVA and CRT from baseline were compared every 6 months up to 3 years (at 6, 12, 18, 24, 30, and 36 months).

BCVA demonstrated a significant logMAR improvement from 0.68 (20/95) to 0.53 ± 0.32 (20/67) (*P* < 0.005) at 6 months and 0.51 ± 0.31 (20/64) (*P* < 0.001) at 12 months. However, no statistical differences were found at 18, 24, 30, and 36 months (*P* > 0.05) (Table 2).

CRT significantly decreased compared to baseline (*P* < 0.001) at 6, 12, 18, 24, 30, and 36 months (Table 2). CRT was also measured using Kaplan-Meier SR analysis after 36 months of follow-up; 74.5% of all patients decreased the CRT compared to baseline during the observational period.

The mean number of ranibizumab injections was 6.98 ± 3.69 [3–20].

At 36 months, Kaplan-Meier SR for the worsening of BCVA was 76.5% (the proportion of patients without a decrease in vision of more than 0.3 logMAR units). According to the number of ranibizumab injections, SR was 88.9% in those who received 7 or more injections versus 69.7% receiving <7 treatments (*P* = 0.12; log-rank test).

**Figure 1:**
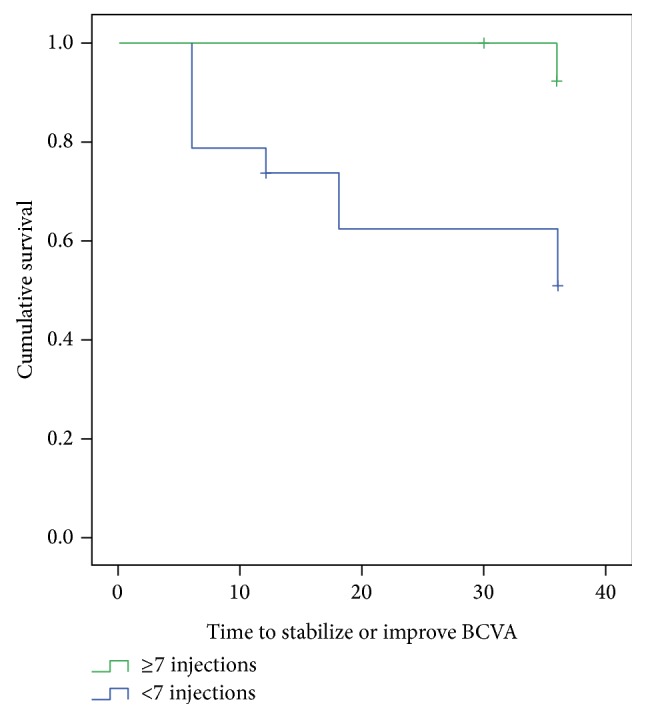
Kaplan-Meier survival plots of stable BCVA (SR was 92.9% in patients who received 7 or more injections versus 51.4% in those treated with <7 treatments (*P* = 0.008; log-rank test)).

The SR calculated for BCVA that remained stable or became better (decrease of 0.01 logMAR units of more) from baseline was 62.7%. According to the number of ranibizumab injections, SR was 92.9% in those who received 7 or more injections versus 51.4% receiving <7 treatments (*P* = 0.008; log-rank test) at 3 years (Figure 1).

## 4. Discussion

To our knowledge of the currently available data, this is the first study to compare retrospective results of treatment with ranibizumab for 3 years according the number of injections. Although the study is limited by its retrospective nature and small number of patients, the lengthy follow-up period (36 months) allowed us to obtain enough data to make meaningful interpretations, especially considering that this study was conducted in normally run vitreoretinal practices, reflecting a “real-life” clinical setting.

The prevalence of wet AMD increases exponentially with aging [9]. This disease causes a great impact on the quality of life, and patients define the consequences of wet AMD as serious as uncontrollable pain or many metastatic or chronic debilitating diseases (severe ischemic transient attack, renal dialysis…). Besides having a devastating effect on the lives of patients, the condition is responsible for a major expense for the economy [10].

Without treatment, exudative AMD causes a significant loss of VA during the first two years of the disease, although most VA loss occurs during the first 3–6 months [11].

One of the primary goals in the management of wet AMD is to halt or delay the progression of visual loss and, if possible, improve VA. The findings of this study confirm the relationship between the number of injections of ranibizumab and the maintenance or improvement of VA. Patients treated with 7 or more injections during the 36-month follow-up showed a better survival rate in both analyses compared with patients who received less than 7 treatments.

Although the SR in worsening BCVA was not statistically significant, there was a clear trend towards a better outcome in the group receiving 7 or more injections (88.9% versus 69.7%, *P* = 0.12).

In the group of stable patients, the difference was even greater and statistically significant (BCVA SR was 92.9% in those who received 7 or more injections of ranibizumab versus 51.4% in those who received <7 treatments, *P* = 0.008).

Recently, Rofagha et al. [12] published the results after 8-year follow-up in 65 patients which participated in the MARINA and ANCHOR [1, 2] trials. To note, 34% worsened their VA (<15 letters) while 43% keep their VA stable or better (≥0 letters) compared to baseline, with a mean number of ranibizumab injections of 6.4 and a mean follow-up of 3.4 years. These results are similar to ours, with a worsening of VA in 23.5% of the patients (>0.3 LogMAR units) whereas 62.7% keep their VA stable or better compared to baseline. They also found that patients who received more intravitreal injections showed better VA outcomes.

In this work, no significant BCVA differences were found at 18, 24, 30, and 36 months (*P* > 0.05), probably because of the insufficient number of injections. Saleh et al. [13] analysed 66 eyes of 60 wet AMD patients treated with ranibizumab with a mean follow-up of 27 months. They did not find a significant VA improvement at the end of the follow-up period (*P* > 0.05) and the percentage of stable patients was 66.6% (compared to 62.7% in our work). Mean number of ranibizumab injections was 5 per year.

The mean number of injections received after 36 months (6.98 ± 3.69) seems to be very low when compared with the previous randomised trials: in the CATT study [14], patients were treated with a mean of 12.6 ± 6.6 ranibizumab injections during 2 years. In the ANCHOR study, the mean number of ranibizumab injections was 21.3 during 24 months of treatment. Such a huge difference in a clinical setting daily routine suggests that although patients are scheduled on a regular basis, the activity of the disease is being undertreated (underestimated) and more aggressive treatment is needed to maintain or improve BCVA during the follow-up.

Unlike this paper, MARINA and ANCHOR [1, 2] trials showed a significant VA improvement after a fixed monthly dosing schedule for intravitreal injections over a two-year period. Despite these impressive results, mostly patients, their families, and clinicians have considerable difficulty to sustain and accept monthly visits, not to mention the economic costs.

Alternate regimens of treatment have evolved in an attempt to provide comparable results in terms of BCVA, but with a fewer number of injections. The “Treat and Extend” (TAE) regimen described by Spaide aims to achieve and maintain a “dry” macula by gradually increasing the length of time between injections in the absence of macular fluid [15]. Growing use of the TAE method with ranibizumab for wet AMD has been described in recent studies with favourable improvements in VA while reducing the number of office visits and injections [16–18]. The 2012 American Society of Retinal Specialists Preferences and Trends survey revealed that the majority of Retinal Specialists members have turned to nonmonthly regimens, with 66.7% using TAE [19].

In conclusion, this study confirms that stable wet AMD patients after 3-year follow-up showed a significantly better BCVA SR (92.9%) if they received 7 or more injections versus those treated with fewer number of ranibizumab injections (51.4%) (*P* = 0.008).

## Figures and Tables

**Table 1:** Demographic and clinical characteristics of the sample.

Age (years)	79.5 ± 7.8
Female (%)	58.8
Right eye (%)	49
High blood pressure (%)	60.8
Pseudophakia (%)	49
Glaucoma (%)	7.8
Type of CNV (FA) (%)	
Classic	48.2
Min. classic	7.4
Occult	44.4
Location of CNV (%)	
Subfoveal	31.8
Juxtafoveal	40.9
Extrafoveal	27.3
IOP (mmHg)	16.16 ± 2.78
BCVA LogMAR (Snellen)	0.68 ± 0.38 (20/95)
CRT (*μ*m)	342.12 ± 121.57
*N*	51

CNV: choroidal neovascularization; FA: fluorescein angiogram; IOP: intraocular pressure; BCVA: best corrected visual acuity; LogMAR: logarithm of the minimum angle of resolution; CRT: central retinal thickness; *N*: number of subjects.

**Table 2:** Comparison of BCVA (LogMAR) and CRT (microns) during the follow-up.

Time (months)	Baseline	6	12	18	24	30	36

BCVA LogMAR (Snellen)	0.68 ± 0.3 (20/95)	0.53 ± 0.3^*^ (20/67)	0.51 ± 0.3^**^ (20/64)	0.59 ± 0.3 (20/77)	0.61 ± 0.3 (20/81)	0.64 ± 0.4 (20/87)	0.67 ± 0.4 (20/93)

CRT (*μ*m)	342 ± 121	297 ± 98^**^	287 ± 94^**^	281 ± 85^**^	288 ± 93^**^	283 ± 92^**^	276 ± 88^**^

Compared to basal value, ^*^ *P* < 0.005; ^**^ *P* < 0.001.

BCVA: best corrected visual acuity; LogMAR: logarithm of the minimum angle of resolution; CRT: central retinal thickness; *N*: number of subjects.
